# Electrical impedance spectroscopy of plant cells in aqueous buffer media over a wide frequency range of 4 Hz to 20 GHz

**DOI:** 10.1016/j.mex.2020.101185

**Published:** 2020-12-17

**Authors:** Kian Kadan-Jamal, Marios Sophocleous, Aakash Jog, Dayananda Desagani, Orian Teig-Sussholz, Julius Georgiou, Adi Avni, Yosi Shacham-Diamand

**Affiliations:** a Department of Materials Science and Engineering, Faculty of Engineering, Tel Aviv University, Tel Aviv 69978, Israel; b Department of Electrical & Computer Engineering, EMPHASIS Research Center, University of Cyprus, Nicosia 1678, Cyprus; c Department of Physical Electronics, School of Electrical Engineering, Faculty of Engineering, Tel Aviv University, Tel Aviv 69978, Israel; d School of Plant Sciences and Food Security, Tel Aviv University, Tel Aviv 69978, Israel; e Thapar Institute of Engineering and Technology, Patiala, Punjab, India

**Keywords:** Electrical impedance spectroscopy (EIS), Vector network analyser (VNA), Plant cells, MSK8, Equivalent circuit

## Abstract

Electrical impedance spectroscopy was performed on suspensions of plant cells in aqueous buffer media over a wide frequency range of 4 Hz to 20 GHz. Custom probes were designed, manufactured, and used for these investigations. Experiments were performed with a custom-made parallel plate probe and impedance analysers in the low-frequency range (4 Hz to 5 MHz), with a custom-made coaxial airline probe and a vector network analyser in the mid-frequency range (100 kHz to 3 GHz), and with a commercial open-ended probe and a vector network analyser in the high-frequency range (200 MHz to 20 GHz). The impedance data acquired were processed in order to eliminate the effects of parasitics and compensate for geometrical differences between the three probes. Following this, the data were fitted to a unified model consisting of the Randles and Debye models. The data were also normalized to a reference measurement, in order to accentuate the effects of cell concentration on the impedance of the suspensions.

• The methodology allows for impedance spectroscopy of cell suspensions over a wide frequency range spanning 10 orders of magnitude.
• It allows for compensation of parasitics and of geometrical variations between probes, using mathematical techniques

## Graphical abstract

**Figure:**
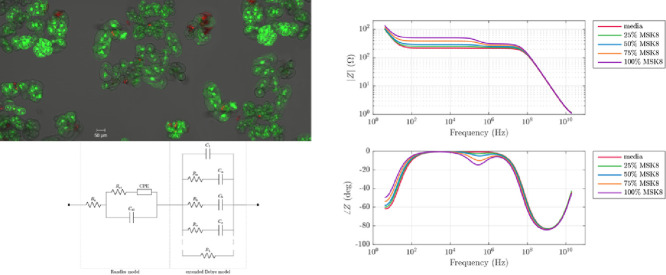

Specifications table

**Table:** 

Subject Area	Engineering
More specific subject area	*Bioelectronics*
Method name	*Electrical spectroscopy of cell suspensions over a wide frequency range*

**Method details**

## Custom probes for use with cell suspensions

In order to perform impedance spectroscopy on the suspension of tomato cells in liquid media, two custom probes were designed and manufactured in-house. The probes were designed to allow for easy insertion and removal of cell suspensions using a standard syringe. The apparatuses were manufactured using non-reactive and long-lasting materials like stainless steel and Teflon.

### Probe for low-frequency measurements

**Fig. 1:**
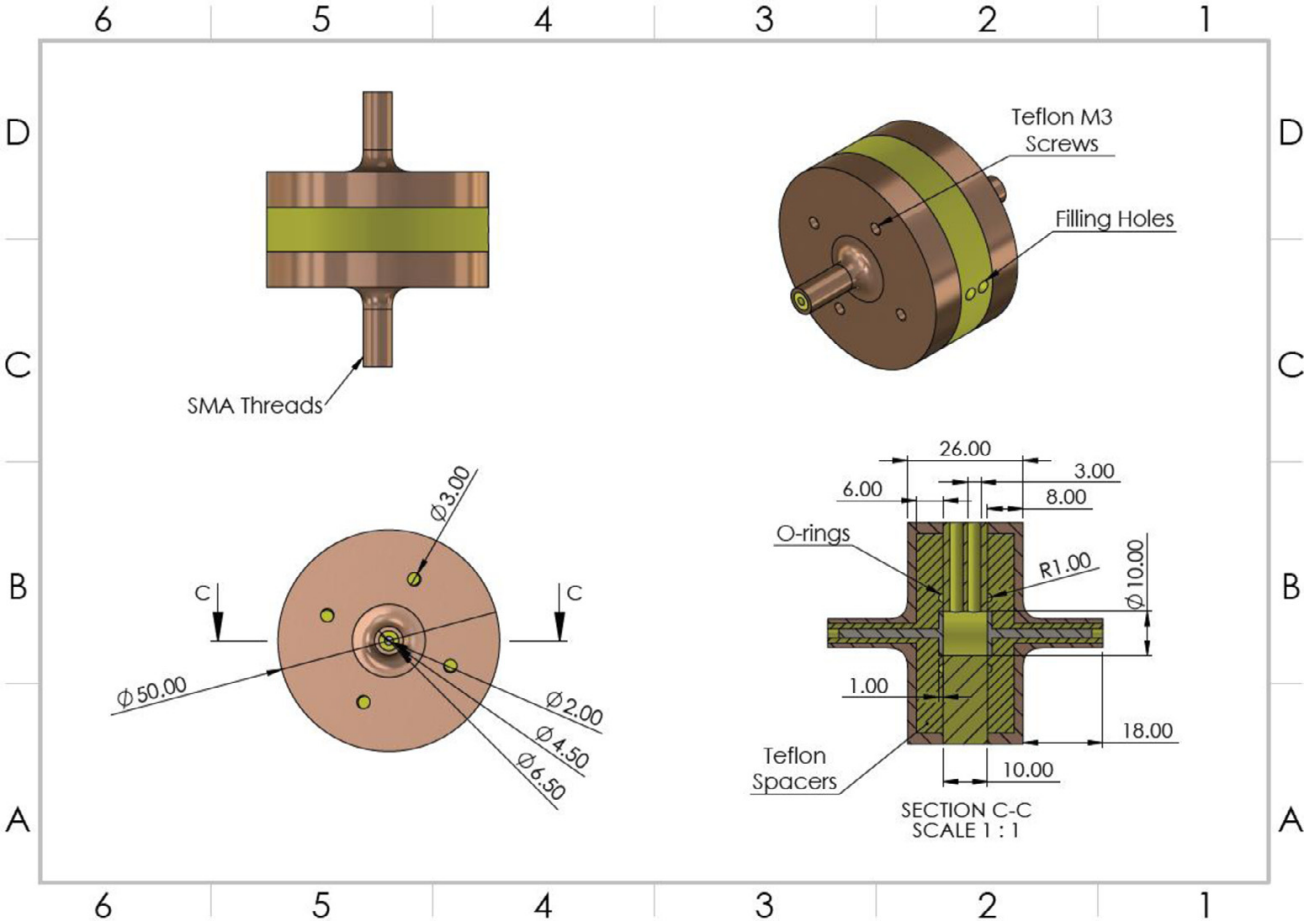
Section and Isometric Views of the Parallel Plate Probe for Low-Frequency Measurements.

In the frequency range of 4 Hz to 5 MHz, the impedance of the cell suspensions was to be measured using an impedance analyzer. Hence, a cylindrical parallel plate design was chosen for this probe. The parallel plates electrodes were made of 316 stainless steel, and the cylindrical walls were made with Teflon. As the outer casing of the probe does not have any contact with the test cavity, it was made of brass. Two O-rings were used to ensure a leak-proof seal between components. M3 Teflon bolts were used to hold the apparatus together, and to ensure a good seal. The volume of the cylindrical cavity was designed to be 700 µl (Fig. 1).

In order to allow for the probe to be connected to the instrument using cables with SubMiniature version A (SMA) connectors, which they are semi-precision coaxial RF connectors, female SMA connectors were incorporated into the probe itself. The stem-like extensions of the parallel plate electrodes were used as the core, and the brass casing was used as the shield with SMA threading on the outer surface of the casing.

### Probe for mid frequency measurements

**Fig. 2:**
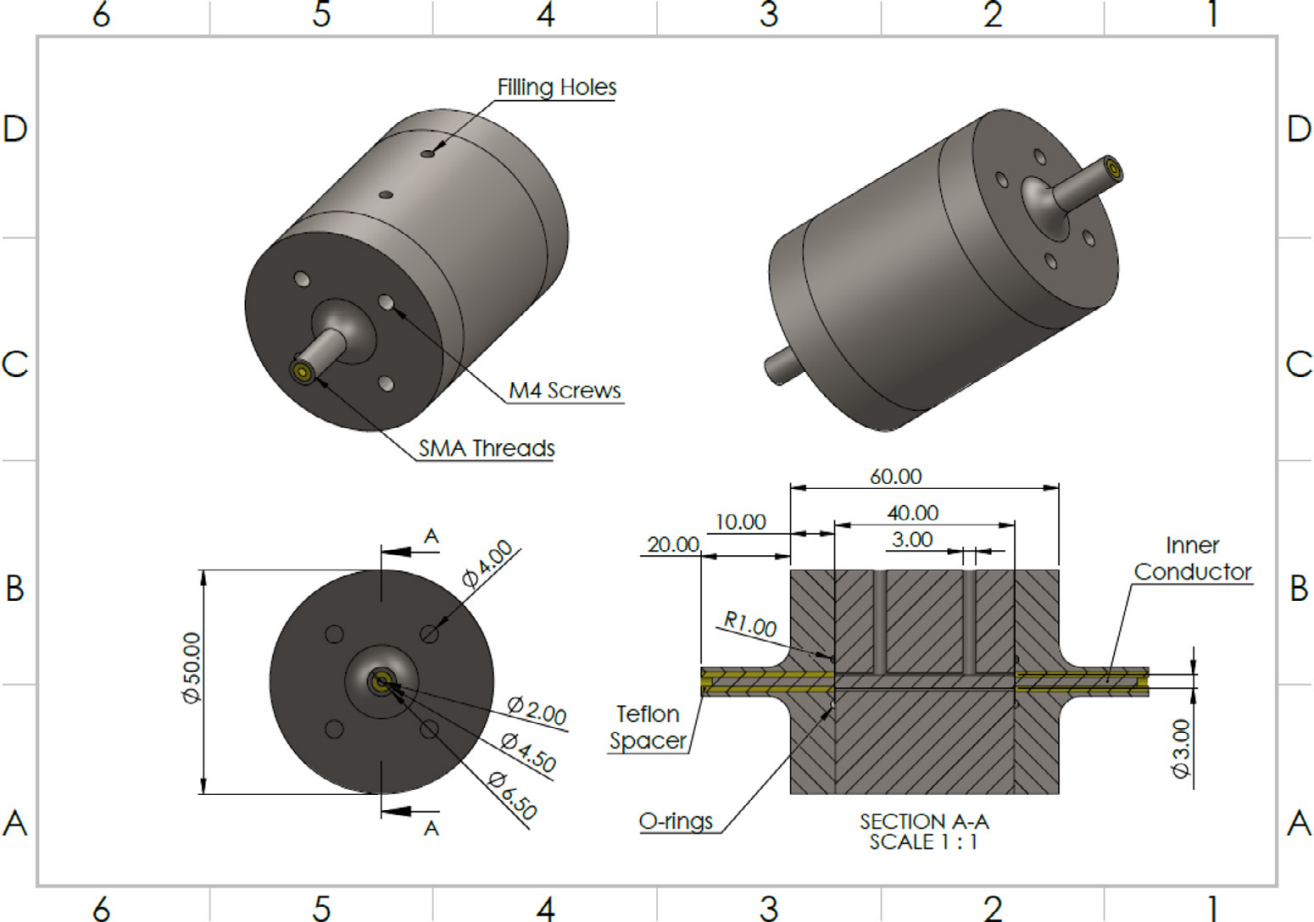
Section and Isometric Views of the Coaxial Airline Probe for Mid Frequency Measurements.

In the frequency range of 100 kHz to 3 GHz, the impedance of the cell suspensions was to be measured using a vector network analyzer. Hence, a coaxial airline design was chosen for this probe. All parts of the probe were made of 316 stainless steel, except for the spacer which was made of Teflon. Two O-rings were used to ensure a leak-proof seal between components. M4 stainless steel bolts were used to hold the apparatus together, and to ensure a good seal. The volume of the coaxial cavity was designed to be 800 µl (Fig. 2).

### Probe for high-frequency measurements

**Fig. 3:**
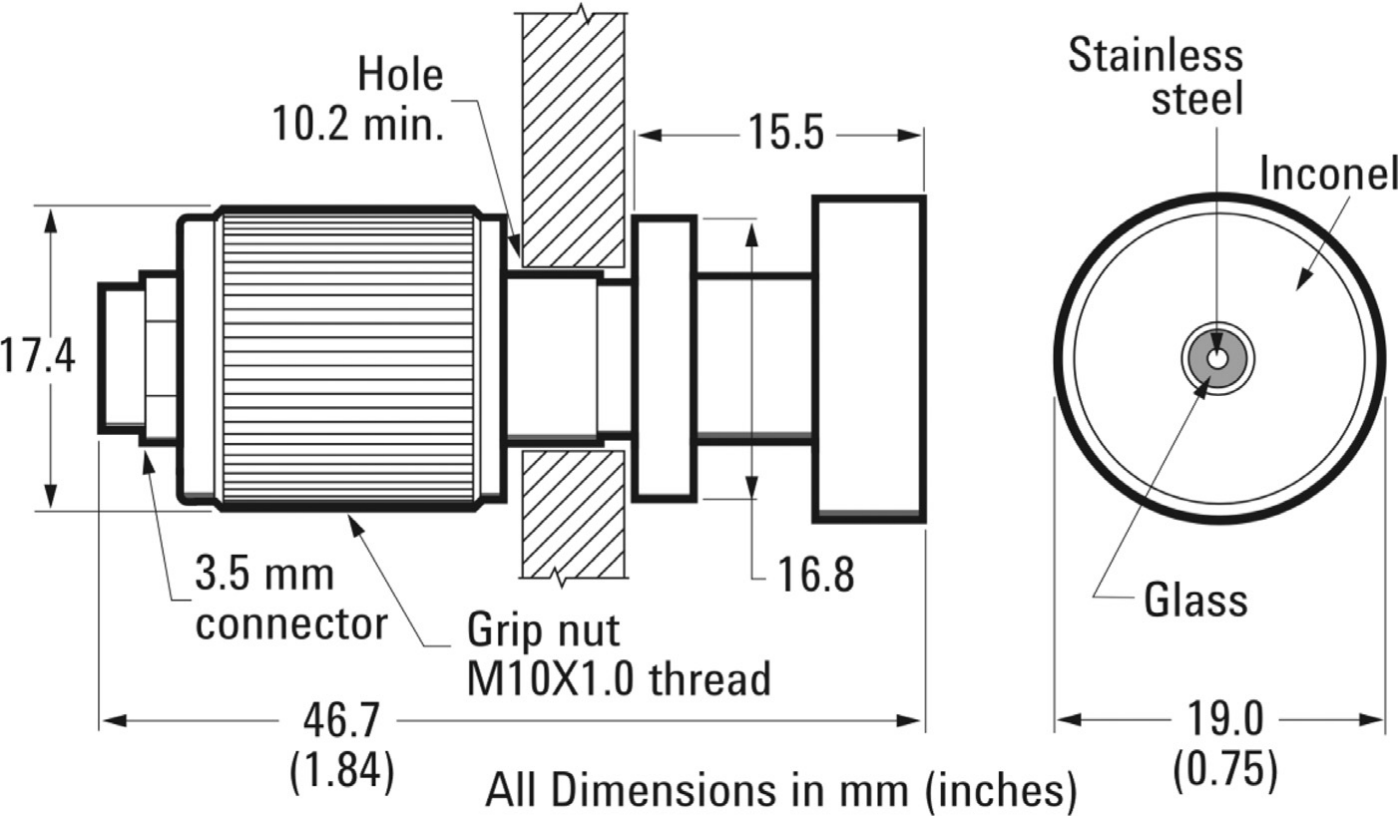
Section Views of the Commercial Open-ended Coaxial Probe for High-Frequency Measurements [1].

In the frequency range of 200 MHz to 20 GHz, a commercial probe from a Keysight 85070E dielectric probe kit was used. The probe was an open-ended coaxial probe, rated for a wide range of temperatures (Fig. 3).

### Cleaning procedure

The probes were deeply cleaned before the first set of experiments. The brass casing of the low-frequency probe and the stainless-steel component of both probes were cleaned in an aqueous solution of 12 gl^−1^ sodium dodecyl sulfate (SDS) and 1 M of sodium hydroxide while being at the boiling point for 8 min. Following this, they were rinsed for a few minutes in hot deionized water followed by cold deionized water. The Teflon parts were cleaned by submerging them in Piranha (H_2_SO_4_·H_2_O_2_(3:1)) solution. As cleaning with Piranha is an exothermic process, the temperature rises to 85 °C. The Teflon parts were maintained at this temperature for 20 minutes and then cleaned with soap and a scrubber. Beyond this initial cleaning, all parts were cleaned using isopropanol (IPA) and deionized water and dried using nitrogen flow, before and after each measurement.

## Experimental setup

### Preparation of cell suspensions

Tomato (S. lycopersicum} cv Mill.; line MSK8 [2]) cell suspension cultures were grown in Murashige and Skoog (MS) medium [3] including vitamins (Duchefa Biochemie), supplemented with 30 gl^−1^ sucrose, 1 mgl^−1^ 2,4-dichlorophenoxyacetic acid (2,4-D) and 0.1 mgl^−1^ kinetin, which was set to pH 5.7. The cell culture was centrifuged at 25 °C in the dark, at approximately 100 rpm. Sub-culturing was performed every two weeks, and the MSK8 cells were used 14–20 days after sub-culturing. Cell concentration cannot be controlled during the growth stage. Hence, a process of centrifugation and decantation was performed in order to obtain a standardized suspension with known cell concentration. Before each experiment, the cells were centrifuged at 1500 rpm for 5 min. The suspension was decanted to obtain a suspension with cell concentration of approximately $1.7\;\times 10^{6}\;ml^{-1}$.This concentrated suspension was then diluted with fresh MS medium to obtain suspensions with lower cell concentration. Thenceforth, the concentration of these diluted suspensions were referred to in terms of the concentration of concentrated solutions. For example, the concentrated suspension was referred to as a 100% solution, whereas a suspension with equal amounts of the concentrated suspension and medium was referred to as a 50% solution.

### Low-frequency measurements

The choice of instruments and probes to be used for experiments depends on the intended frequency range of operation. Parallel plate probes are easy to manufacture and use with impedance analyzers. Their simple topology also allows for easier modelling. However, they are not ideal for use with frequencies above a few MHz, due to reflections and sensitivity to high-frequency noise. Additionally, the parasitics due to the thick Teflon walls dominate at these frequencies. Above this range of frequencies, it is necessary to use a vector network analyzer (VNA), with a coaxial probe. Such probes include coaxial airline probes (with the topology of a transmission line) and open-ended probes. Of these, coaxial airline probes are suitable for frequencies up to a few GHz and open-ended probes must be used for higher frequencies [4]. In the frequency range of 4 Hz to 5 MHz, the measurements were carried out with the parallel plate probe connected to a Hioki 3570 impedance analyzer. The probe was connected to the impedance analyzer using SMA cables and SMA to "Bayonet Neill–Concelman" (BNC) – a miniature quick connect/disconnect RF connector used for coaxial cable adapters. An impedance analyzer was used in this frequency range as it reliably provided direct impedance measurements, without the need for additional data processing. The cylindrical test cavity of the low-frequency probe was filled with the cell suspension using a 1 ml syringe with a micro-needle. The measurements were performed by supplying 0.5 V (RMS) sinusoids. A variety of actuation magnitudes were tested, and this particular magnitude was chosen as it yielded a good signal to noise ratio and did not have the potential to trigger undesirable chemical reactions or movement of cells due to electrical field gradients. The frequency range of 4 Hz to 5 MHz was covered in a logarithmic sweep of 801 points. The measurements were repeated multiple times, yielding results well within the measurement error margins of the equipment used (±0.08% for |Z|, ±0.05° for ∠Z). The sedimentation time of the cell suspension was separately observed and was found to be much longer than the time taken to perform the measurements. Hence, a shaking/stirring system was not used.

### Mid frequency measurements

In higher frequencies, i.e. above 10 MHz, vector network analyzers are preferred for impedance analysis. In such cases, the scattering parameters (S-parameters) need to be measured and then converted to equivalent impedance parameters (Z-parameters). In the frequency range of 100 kHz to 3 GHz, the measurements were carried out with the coaxial airline probe connected to an Agilent E5061B vector network analyzer. The probe was connected to the VNA using SMA cables. The scattering parameters of the setup were measured, and the equivalent impedance parameters were calculated using standard transformations [5]. Thus, the resulting Z_11_ was used as a measure of the impedance of the solution (combined with the probe parasitics which were mathematically removed later). At the beginning of each set of experiments, calibration was performed using a Keysight 85562A calibration kit (core diameter 2.92 mm DC to 40 GHz), following a standard Short, Open, Load, Through (SOLT) calibration procedure [6]. The coaxial test cavity of the coaxial airline probe was filled with the cell suspension using a pipette. The frequency range of 100 kHz to 3 GHz was covered in a logarithmic sweep of 1601 points. The measurements were repeated multiple times, yielding very similar results every time. Hence, the variation between measurements under identical conditions was less than the accuracy of the vector network analyzer (±1.5 dBm for the S-parameters). As with the low-frequency measurements, a shaking/stirring system was found to be unnecessary and hence not employed.

### High-frequency measurements

In the frequency range of 200 MHz to 20 GHz, the measurements were carries out with a Keysight 85070E dielectric probe kit, connected to an Agilent E8361C vector network analyzer (VNA). At the beginning of each set of experiments, calibration was performed using the calibration kit provided with the probe kit, following a standard Short, Open, Load (SOL) calibration procedure. As instructed in the probe kit's instruction manual, deionized water was used as the reference load. The cell suspension was kept in a small plastic cup of 3.5 cm diameter kept on a plastic base. The cup and the base were placed on a moving stage and raised vertically bringing the suspension in contact with the dielectric probe. The scattering parameters of the setup were acquired from the VNA, and converted to the equivalent dielectric spectra, including both permittivity $\varepsilon^{\prime }$and dielectric loss $\varepsilon^{\prime \prime }$. This conversion was performed using the software provided by the manufacturer of the probe kit, using the Debye model of dielectric relaxation. The frequency range of 200 MHz to 20 GHz was covered in a logarithmic sweep of 201 points. The measurements were repeated multiple times, yielding very similar results every time. Hence, the variation between measurements under identical conditions was less than the accuracy of the vector network analyzer (±1.5 dBm for the S-parameters). As with the low and mid-frequency measurements, a shaking/stirring system was found to be unnecessary and hence not employed.

## Data processing

### Modelling and removal of parasitics

There is a clear need for more data and models unifying the dielectric properties of biological materials in the low-frequency (up to a few kHz), the medium-frequency (up to a few hundreds of MHz), and the high-frequency ranges (up to 20 GHz). The necessity of such models and the frequency ranges of interest have been previously discussed [7]. A unified model consisting of a series connection of the Randles model for electrode/electrolyte interfaces and the Debye model for dielectric relaxation was used to model the cell suspension [8]. Although the data processing methodology is described for this unified model, it can be used for any equivalent circuit model with minor modifications.

**Fig. 4:**
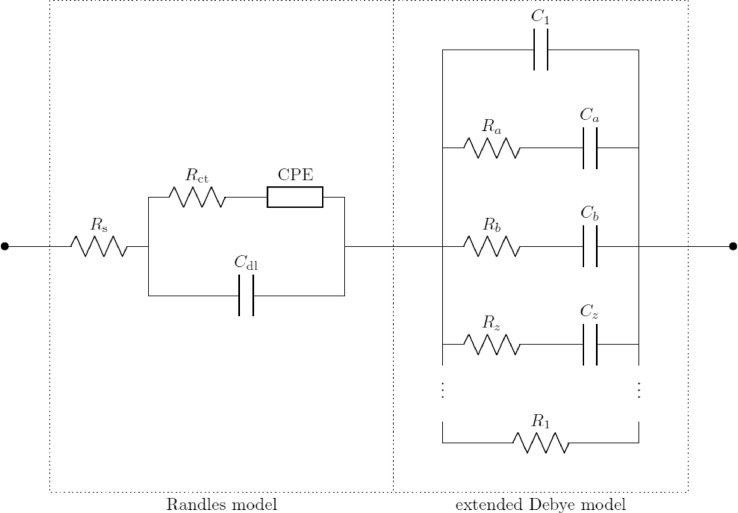
Unified Equivalent Circuit Model of Cell Suspensions in Aqueous Buffer Media.

**Fig. 5:**
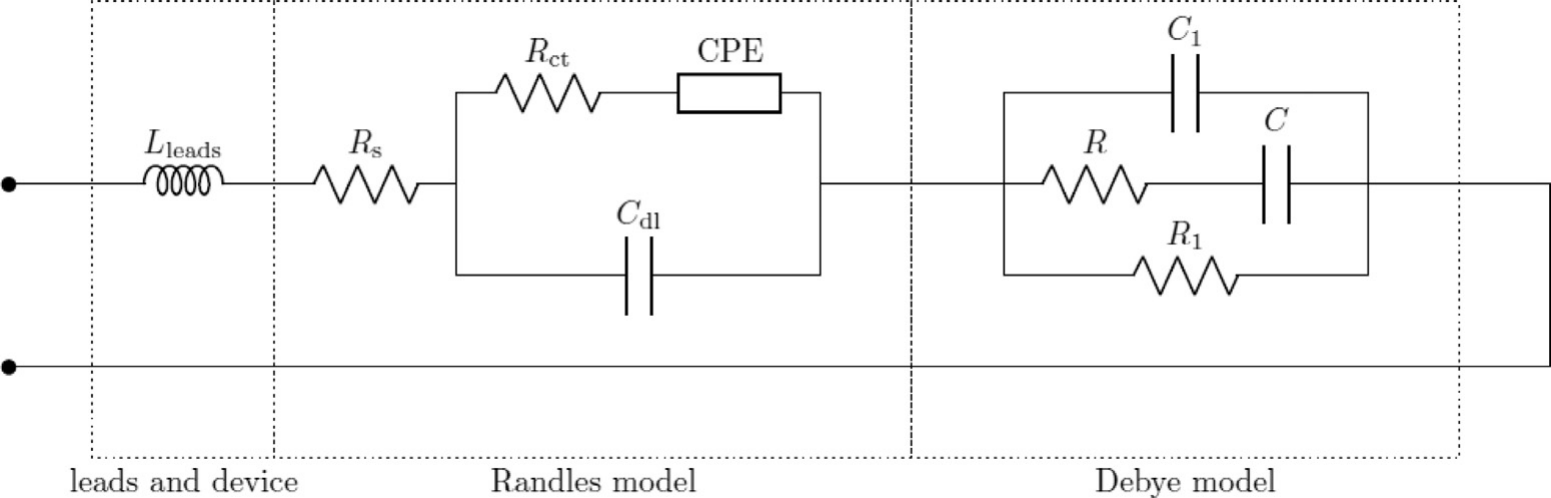
Effective Circuit Model Used to Model Impedance Behaviour in the Low Frequency Range.

**Fig. 6:**
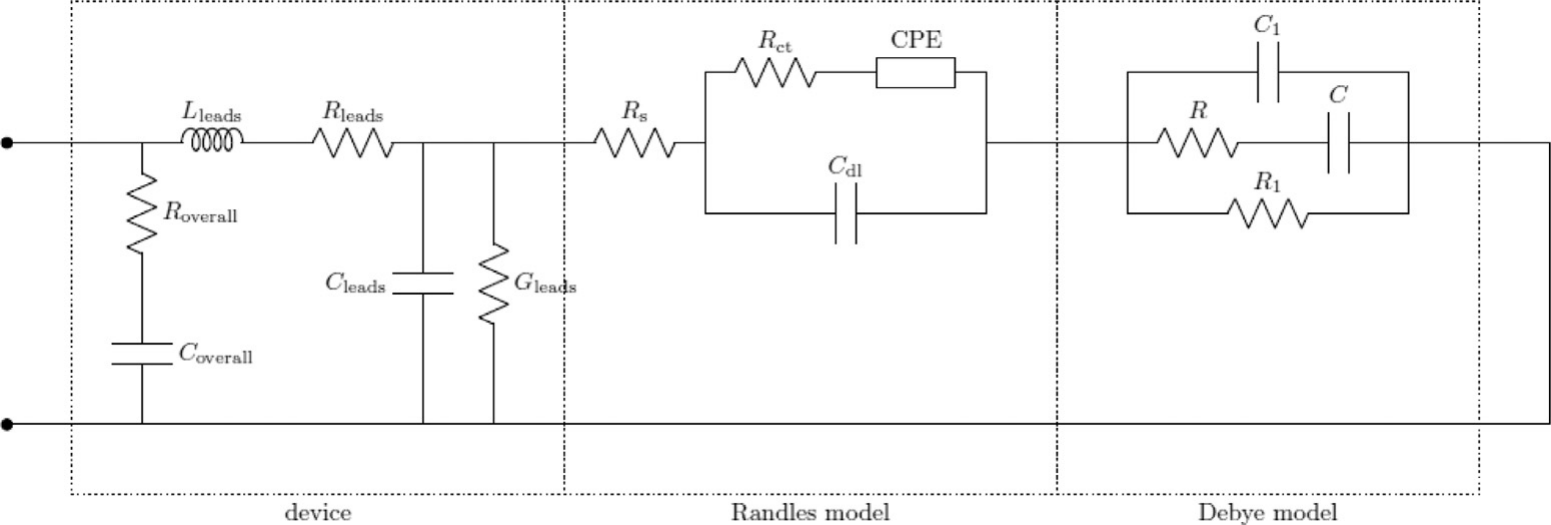
Effective Circuit Model Used to Model Impedance Behaviour in the Mid Frequency Range.

**Fig. 7:**
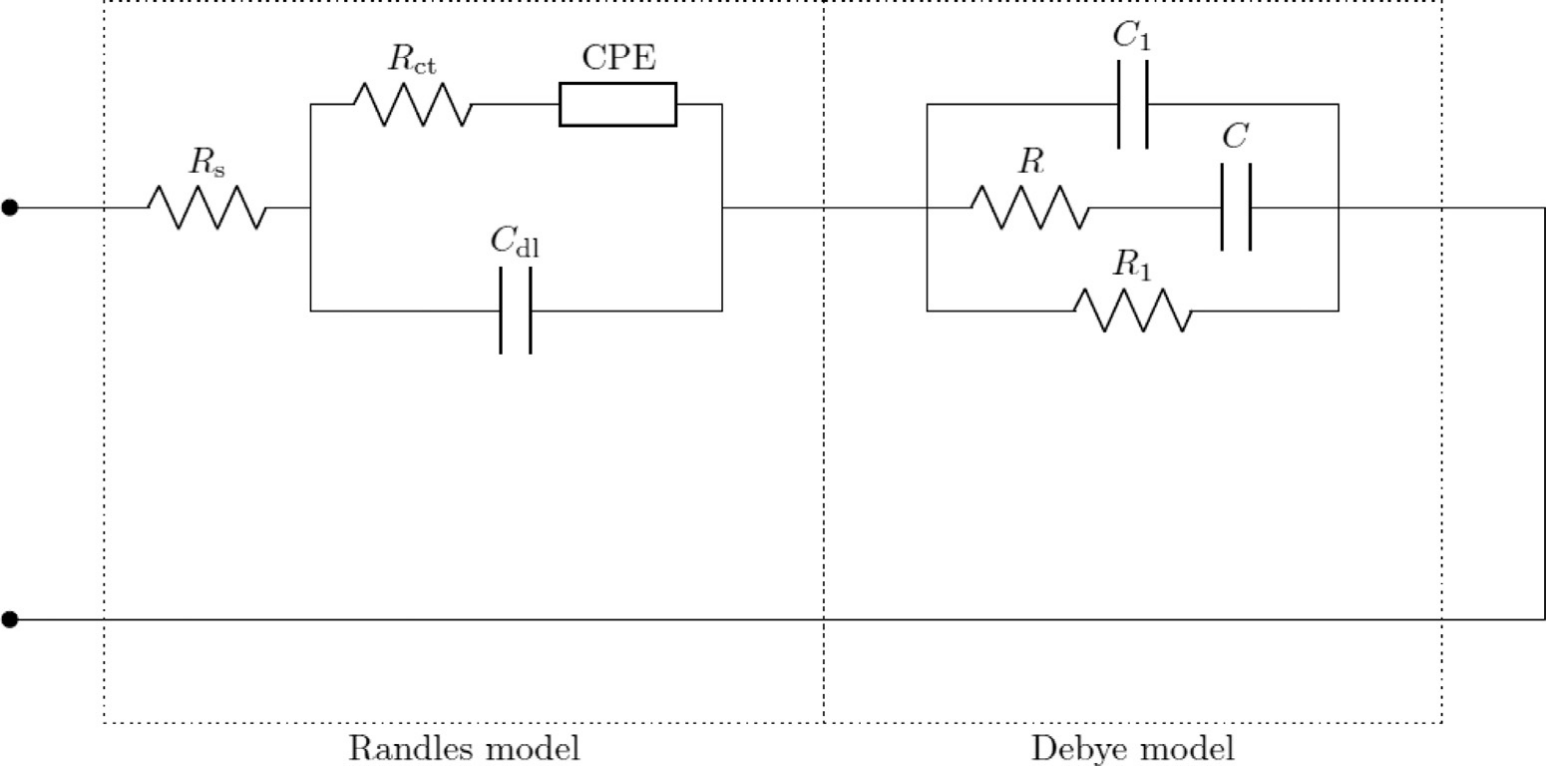
Effective Circuit Model Used to Model Impedance Behaviour in the High-Frequency Range.

Each pair (R_a_, C_a_), …, (R_z_, C_z_) in Fig. 4 represents a loss mechanism due to the presence of cells in the suspension. As the suspension used for these investigations consisted of a single type of cells in a single aqueous medium, a single loss mechanism was taken into consideration. Thence, the corresponding resistance and capacitance were denoted by R and C instead of R_a_ and C_a_. The unified equivalent circuit model has been discussed in depth in previous works [7]. In addition, the apparatuses used in each frequency range had parasitics, which were added to the model used for fitting. These components were connected in a topology based on the geometry of the probes and the electrical connections of the setup. The effective models used in the three frequency ranges are shown in Figs. 5–7. For the low-frequency setup, a simple inductor was used to model the cables connecting the impedance analyzer to the probe. This simple model sufficiently represents the parasitics of SMA cables up to a few MHz. For the mid-frequency setup, as the probe used was a coaxial airline probe, the simplest transmission line model with an extra resistor and capacitor in parallel was used to model the probe and the cables. This is a widely accepted model [5] of a coaxial transmission line, representing the material and topological parasitics. This model is the simplest lumped approximation of a distributed transmission line and can be improved by adding more such lumped elements in series. However, as a first approximation to study the overall impedance spectra of cell suspensions, this simple model suffices. In the high-frequency setup, all parasitics are removed by the probe kit's software using data collected from the calibration. Hence, the effective circuit model is the unified model itself.

The measured impedance spectra from each frequency range were fitted to the corresponding model, using a standard least square fitting algorithm based on the magnitude spectra. The effective impedance spectra of the cell suspensions were then computed, ignoring the parasitics and taking into account the Randles and Debye models only, as expressed in Eq. (1).

(1) $$Z_{eff}\left(\omega \right)=R_{s}+\left(R_{ct}+Z_{W}\right)∥\frac{1}{j\omega C_{dl}}+\frac{1}{j\omega C_{1}}∥\left(R+\frac{1}{j\omega C}\right)∥R_{1}$$

where ω is the angular frequency, Z_W_ is the impedance of the Warburg element in the Randles model, and the other parameters are as described in the unified model (Fig. 4). The results and the efficacy of the fitting methodology have already been discussed in depth [7].

### Compensation for geometrical differences

The experimental setups used in the three frequency ranges were different in terms of geometry and topology. Hence, it was necessary to introduce multiplicative factors to compensate for these differences. As modelling a cylindrical parallel plate topology is easier than coaxial and half-unbounded topologies, the low-frequency setup was used as the reference. The impedance spectra of the mid-frequency range were multiplied by a compensation factor, such that the impedance gains of both sets of measurements matched up at 5 MHz, i.e.

(2) $${\hat{Z}}_{mid}\left(f\right)=Z_{mid}\left(f\right)\cdot \left|\frac{Z_{mid}\left(f=5MHz\right)}{Z_{low}\left(f=5MHz\right)}\right|$$

where $Z_{low}\left(f\right)$represents the impedance spectrum in the low-frequency range, $Z_{mid}\left(f\right)\;$represents the impedance spectrum in the low-frequency range, and ${\hat{Z}}_{mid}\left(f\right)$ represents the impedance spectrum in the low-frequency range after compensation. Similarly, the impedance spectra of the high-frequency range were factored to match the mid-frequency spectra (after compensation w.r.t low-frequency spectra) at 200 MHz, i.e.

(3) $${\hat{Z}}_{high}\left(f\right)=Z_{high}\left(f\right)\cdot \left|\frac{Z_{high}\left(f=200MHz\right)}{Z_{mid}\left(f=200MHz\right)}\right|$$

where ${\hat{Z}}_{mid}\left(f\right)$represents the (compensated) impedance spectrum in the mid-frequency range, $Z_{high}\left(f\right)$represents the impedance spectrum in the high-frequency range, and ${\hat{Z}}_{high}\left(f\right)$ represents the impedance spectrum in the high-frequency range after compensation. After the inclusion of these compensation factors, all impedance spectra were considered to be equivalent to impedance spectra for the geometry of the low-frequency setup, i.e. a cylindrical parallel plate topology with diameter 1 cm and length 1 cm. As the frequency ranges used to have significant overlap (100 kHz to 6 MHz and 200 MHz to 3 GHz), there exists duplicity of data in these parts of the frequency spectrum. The impedance values were observed to be identical - within a reasonable range of error - thus validating the compensation procedure and reaffirming that differences in probe topologies do not affect the impedance characteristics of cell suspensions.

### Modelling for extraction of equivalent circuit parameters

An important assumption in the compensation procedure and the equivalence conclusions described in 3.2. is that the impedance spectra must be that due to the cell suspensions only. This assumption only holds once the parasitics have been modelled and removed, following the procedure described in 3.1. Similarly, the equivalent circuit parameters corresponding to the cells and media extracted when performing fitting for parasitic removal are not valid once the compensation procedure is performed. Hence, in order to obtain equivalent circuit parameters, the following steps must be performed in orde:

1. Fit the measured impedance spectra to the corresponding circuit models (including cells and media, and parasitics).
2. Compensate for geometrical differences (i.e. ``stitch'' the spectra from different ranges together).
3. Fit the processed spectra to the unified model (cells and media only).

This process was performed and the equivalent circuit parameters corresponding to the unified model (Fig. 4) were extracted. Of these, the charge transfer resistance (R_ct_) and the impedance of the Warburg element (Z_CPE_) dominate in the very low frequencies. As our investigation covered the frequency spectrum down to 4 Hz only, the effects of these were not evident. Hence, when the spectra were fitted to the model, these parameters converged to sufficiently large values and did not vary with cell concentration. The fitting would be equally valid for a wide range of values of these parameters, and hence it was impossible to confidently extract exact values of these parameters. Due to this fact, and as the values of these parameters were inconsequential to the observed impedance spectra, their correlation with cell concentration was not studied.

### Normalization of impedance

In order to investigate the effect of cell concentration on impedance spectra, the change in the equivalent circuit parameters can be studied. However, since the change in impedance due to these are small in comparison with the impedance itself, it is helpful to accentuate these effects for improved visual perception. To do so, the impedance spectra were normalized with respect to the impedance spectra of the medium without cells. This was done by a standard normalization transformation, i.e.

(4) $$Z_{cells,\;normalized\;}\left(f\right)=\frac{Z_{cells\;}\left(f\right)}{Z_{medium\;}\left(f\right)}$$

where $Z_{cells\;}\left(f\right)$ is the impedance spectrum of the cell suspension, and $Z_{medium\;}\left(f\right)$ is the impedance spectrum of the medium without cells. Following this normalization, the data can be presented in the form of a Bode plot, with

(5) $$\left|Z_{cells,\;normalized\;}\left(f\right)\right|=\frac{\left|Z_{cells\;}\left(f\right)\right|}{\left|Z_{medium\;}\left(f\right)\right|}$$

(6) $$∠Z_{cells,\;normalized}\left(f\right)=∠Z_{cells}\left(f\right)-∠Z_{medium}\left(f\right)$$

## Declaration of Competing Interest

The authors declare that they have no known competing financial interests or personal relationships that could have appeared to influence the work reported in this paper.
